# Undiagnosed hypertension in Peru: analysis of associated factors and socioeconomic inequalities, 2019

**DOI:** 10.1016/j.heliyon.2021.e07516

**Published:** 2021-07-09

**Authors:** Delia Vanessa Guerrero-Díaz, Akram Hernández-Vásquez, Wency Cecilia Montoya-Rivera, Carlos Rojas-Roque, Manuel Alberto Chacón Díaz, Guido Bendezu-Quispe

**Affiliations:** a Universidad Científica del Sur, Lima, Peru; b Centro de Excelencia en Investigaciones Económicas y Sociales en Salud, Vicerrectorado de Investigación, Universidad San Ignacio de Loyola, Lima, Peru; c Universidad de Buenos Aires, Buenos Aires, Argentina; d Instituto Nacional Cardiovascular, EsSalud, Lima, Peru; e Universidad Privada Norbert Wiener, Centro de Investigación Epidemiológica en Salud Global, Lima, Peru

**Keywords:** Hypertension, Risk factors, Healthcare disparities, Chronic disease, Peru

## Abstract

**Objective:**

To determine the factors associated and measure the socioeconomic inequalities in people with undiagnosed hypertension in Peru.

**Materials and methods:**

An observational, cross-sectional, analytical study was performed using data from the 2019 Demographic and Family Health Survey (ENDES, acronym in Spanish) database. The dependent variable was the presence of undiagnosed hypertension (mean systolic blood pressure ≥140 mmHg and/or mean diastolic blood pressure ≥90 mmHg in the two blood pressure measurements and with no prior diagnosis of hypertension by a health care professional). Adjusted prevalence ratios were estimated to determine the factors associated with undiagnosed hypertension. The socioeconomic inequality in undiagnosed hypertension was estimated using concentration curves and the Erreygers concentration index.

**Results:**

67.2% of 3697 persons with hypertension had not been diagnosed. Non-diagnosis of hypertension was more prevalent in men who were residents of the Coast and in inhabitants residing at more than 3000 m above sea level. Being 50 years of age or older, having health insurance, being obese and having diabetes mellitus were associated with a lower prevalence of undiagnosed hypertension. Inequality of the non-diagnosis of hypertension was found to be concentrated in the poorest population.

**Conclusions:**

At least one out of every two adult Peruvians with hypertension have not been diagnosed with this condition. Socioeconomic inequality was found, as well as socio-demographic and health-related factors associated with undiagnosed hypertension. Our findings identify some population subgroups in which interventions for screening and treatment of hypertension should be prioritized in order to reduce both inequalities and complications of hypertension among the most vulnerable.

## 1 Introduction

Hypertension is the most important risk factor for cardiovascular mortality worldwide [1] and is responsible for 45% and 51% of deaths by ischemic cardiopathy and cerebrovascular accident, respectively [2]. Moreover, hypertension affects more than one trillion people [3] and produces nine million deaths annually [2], making it a public health concern that needs to be timely diagnosed and treated [3].

Early diagnosis of hypertension is the first step to achieve adequate control of blood pressure. It is estimated that in one out of every five people with hypertension, the condition is controlled [4], and among patients not diagnosed with hypertension, 3 out of every four are considered to be at high cardiovascular risk [5]. There is a high prevalence of individuals with undiagnosed hypertension (66%), ranging from 50.4% to 59.9%, mainly in middle-income and in Southeast Asian countries [6, 7, 8, 9], which contrast with the prevalence of between 4.1% and 6.5% in the United States [10]. According to the Cardiovascular Risk Factor Multiple Evaluation in Latin America (CARMELA) study, the prevalence of undiagnosed hypertension varies between 24% and 47% in 7 cities of Latin America [11]. Thus, undiagnosed hypertension is a challenge in Latin American countries (which are the most affected by this condition).

Previous studies have reported the following factors as being associated with undiagnosed hypertension: younger age, male sex, living in a rural area, having a lower level of education, belonging to a low socioeconomic level, not having health insurance, and not having overweight or obesity [7, 8, 9, 12]. These factors lead to a delay in the treatment of hypertension and, consequently, an increase in cardiovascular complications [1, 7]. The promotion of primary health prevention based on education and healthy lifestyles is necessary to decrease the burden of hypertension and premature mortality due to cardiovascular conditions as well as reduce the inequalities in undiagnosed hypertension based on individual characteristics such as socioeconomic and educational level [4, 7, 8].

Peru is currently undergoing an epidemiologic transition, with an increase of cases and deaths due to non-communicable chronic diseases, among which cardiovascular diseases are second as the cause of death in the last years [13]. According to the Demographic and Family Health Survey of 2019 (ENDES 2019), in Peru, 10.2% of people aged 15 or older have been diagnosed with hypertension by a medical professional [14]. However, there is no evidence of the prevalence of undiagnosed hypertension, its associated factors, and socioeconomic inequalities in Peru. It is imperative to identify these factors in order to create strategies for the most vulnerable populations and thereby reduce the burden of hypertension and its complications. Consequently, the objective of this study was to determine the prevalence of undiagnosed hypertension, the factors associated with its appearance, and the socioeconomic inequalities related to its diagnosis.

## 2 Materials and methods

### 2.1 Design of the study

We conducted an observational, cross-sectional, analytical study using the ENDES 2019 database. ENDES is a nationally representative survey compiled by the National Institute of Statistics and Informatics of Peru (INEI). The ENDES sampling is two-stage, probabilistic, stratified, and independent at the departmental level and by urban/rural area. The primary sampling unit is constituted by clusters selected by probability proportional to their size. The secondary sampling unit is constituted by the dwellings selected by balanced sampling using the variables boys and girls under the age of five years and women of childbearing age. The ENDES sample size corresponds to 3,254 clusters equivalent to 36,760 dwellings. Any additional methodological details of the ENDES can be consulted in the ENDES final report [15].

### 2.2 Sample

**Figure 1:**
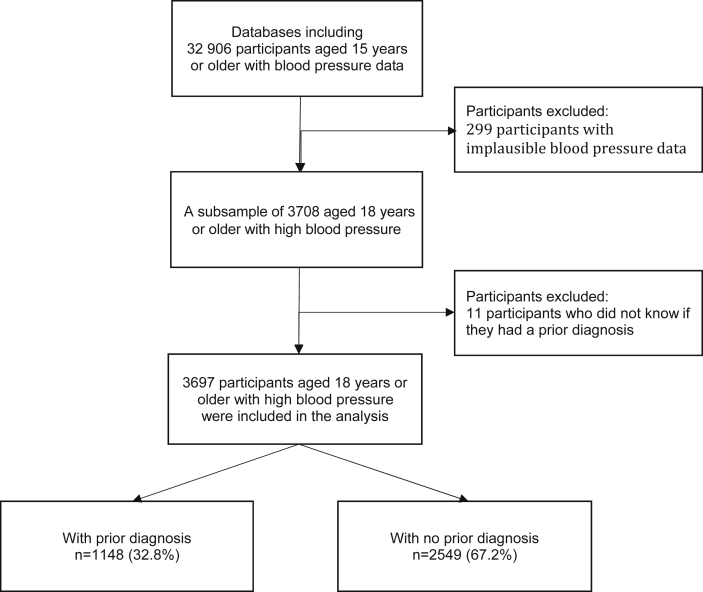
Flowchart of the participants included in the study analysis.

The ENDES study sample for the health questionnaire includes 32,906 individuals in whom blood pressure data is available from individuals greater than 15 years of age who are habitual residents of the selected dwellings in urban and rural areas. In order to only include plausible blood pressure measurements, a cut-off point previously used in other studies was used: systolic blood pressure (SBP) < 270 mmHg and >70 mmHg and diastolic blood pressure (DBP) < 150 mmHg and >50 mmHg. Data including SBP or DBP measurements with no plausible values were omitted from the analyses [16, 17]. After excluding participants with no plausible blood pressure data (n = 299) and individuals who did not know if they had a prior diagnosis (n = 11), the study included 3697 individuals aged 18 or more with blood pressure values compatible with hypertension at the time of the survey (Figure 1).

### 2.3 Dependent variable

The dependent variable was the presence of undiagnosed hypertension (yes/no), constructed from the survey data. The presence of undiagnosed hypertension was defined if, at the time of the survey, a person had a mean SBP value ≥140 mmHg and/or a mean DBP ≥90 mmHg in the two blood pressure measurements and that hypertension had not been previously diagnosed by health personnel such as a doctor or nurse. Otherwise, it was defined as the absence of undiagnosed hypertension. The first measurement was taken after a rest period of five  minutes and the second measurement was taken 2 min after the first measurement [18].

### 2.4 Independent variables

The following independent variables were considered: age in years (18–29/30-49/50–69/70 or more), gender (female/male), ethnicity (non native, native, afro-peruvian), educational level (none or pre-school/primary education/secondary education/higher education), marital status (single/married/widowed, divorced, or separated), health insurance (yes/no), body mass index (underweight or normal/overweight/obesity), physical or psychological limitation (yes/no), depressive symptoms (determined by a score of ten or more in the Patient Health Questionnaire-9 [PHQ-9] screening test in the 14 days prior to the survey [yes/no]), diabetes (yes/no), smoking status (if smoked cigarettes in the last 30 days [yes/no]), binge drinking (consumption of five and four or more alcoholic beverages on the same occasion for men and women, respectively, in the last 30 days prior to the survey [yes/no]), wealth quintile (richest/rich/middle/poor/poorest), area of residence (urban/rural), natural region of residence (Jungle/Andean/Coast), and altitude in meters above sea level (MASL) The selection and inclusion of these independent variables in the study were based on a literature review [7, 8, 9, 12].

### 2.5 Statistical analysis

All statistical analyses were performed using Stata v.14.2 software (Stata Corporation, College Station, Texas, USA). The characteristics of the ENDES sampling were specified using the command “*svy*”, which includes weighting according to strata, weighting factor, and design. In addition, the *subpop* option was used to make estimates of the subpopulation (adults aged 18 years or more with hypertension).

In order to characterize the study population, univariate analyses were performed using simple frequencies and weighted relative frequencies. To standardize the prevalence of undiagnosed hypertension, the ages of the reference population according to the World Health Organization (WHO) were used [19]. Standardization of the prevalence of undiagnosed hypertension estimates an age-adjusted prevalence that is a weighted average for each population to be compared. Differences between proportions were evaluated using the chi-square test.

Generalized linear models of the Poisson family and the log-link function were estimated to determine the factors associated with undiagnosed hypertension. At first, a bivariate regression model was estimated to obtain crude prevalence ratios together with their 95% confidence intervals (CI). Subsequently, with the factors which obtained a value of p <0.20, a multivariate model was estimated to obtain adjusted prevalence ratios (aPR) together with their 95% CI. Statistical significance was considered in all estimates if the p-value was less than 0.05. Multicollinearity of the independent variables was evaluated using the variance inflation factor (VIF) using the command "*collin*". For all the independent variables analyzed, the VIF values obtained were less than 2.5, which indicates the absence of multicollinearity problems [20].

A sensitivity analysis was conducted to identify the factors associated with undiagnosed hypertension. This analysis was restricted to the second measurement because of environmental and psychological factors that could influence the value of the first blood pressure measurement [21]. This analysis was aimed at evaluating the potential effect of changes in the consistency and plausibility of measurements.

In order to measure the socioeconomic inequalities in undiagnosed hypertension, concentration curves (CC) and Erreygers concentration indices (ECI) were estimated. Following O'Donnell *et al.* [22], CC describes the relationship between the cumulative percentage of the population, ordered by the level of wealth (X-axis), and the cumulative percentage of undiagnosed hypertension (Y-axis) with the diagonal line of equality (line of 45°). The inequality is estimated according to the concavity or convexity of the curve. The further the CC moves from the equality line, the greater the degree of inequality. If the CC is below the equality line, there is a greater burden of undiagnosed hypertension in the wealthiest population. When the CC is above the equality line, it indicates a higher burden of undiagnosed hypertension among the poorest.

On the other hand, using the command “conindex [23]”, the ECI was estimated because the dependent variable has a dichotomous nature [23, 24]. Mathematically, the ECI is obtained by the formula:

$$ECI\left(y\right)=\frac{1}{n}\sum_{i=1}^{n}\left[\frac{4a_{i}}{\left(a^{max}-a^{min}\right)}\left(2R_{i}-1\right)\right]$$

in which *y* is the dependent variable, $a_{i}\exists \left(a^{max}-a^{min}\right)$ denotes the dichotomous variable with limit values 0 and 1, $\left(2R_{i}-1\right)$ denoting the fractional range of the wealth level. For any ECI, the values it takes range between -1 and 1, which reflects the variability and strength of the relationship between the variables studied. Positive (negative) values reflect a higher charge of undiagnosed hypertension in the wealthiest (poorest) population. The differences in ECI according to the different population subgroups were performed using the z-test when two groups were compared and the f-test when three or more groups were compared. Comparisons were made assuming a large sample size, and thus, the assumption of the equality of variances was relaxed.

### 2.6 Ethical considerations

The study did not require the approval of an ethics committee as it is an analysis of secondary aggregated data that is in the public domain and does not allow the identification of the participants evaluated.

## 3 Results

We analyzed data from a total of 3697 adults with blood pressure values compatible with hypertension. The age group of 50–69 years old represented the highest proportion of the population studied (39.5%), while the age group of 18–29 years was the least prevalent (8.0%). According to sex, 60.5% were male. Among the respondents, 24.1% identified themselves as native and 10.6% as afro-peruvians. Concerning marital status, 62.4% were married, and only 11.4% were single. In relation to the area of residence, 84.3% lived in an urban area, and with respect to the geographic region, 70.7% resided on the Coast. Most respondents resided at between 0 to 499 MASL (72.9%), and only 10.7% resided at more than 3000 MASL. Regarding educational level, most had a high school education (35.1%), and only 7.1% had none or preschool education. Most of the individuals had health insurance (74.4%), and 23.7% were in the richest wealth quintile. Overweight (42.5%) or obesity (36.5%) accounted for 79% of the individuals, 4.1% presented a physical or psychological limitation, 7.8% presented depressive symptoms, and 9.4% reported having diabetes mellitus. In regard to consumption patterns, 12.1% smoked, and 24.6% presented episodes of excessive alcohol consumption (Table 1).

**Table 1:** Characteristics of the adults included in this study (ENDES 2019).

Characteristic	Absolute frequency (n = 3697)	%∗ (95% CI)
Age (mean/SD)	56.7 (15.5)	
Age group (years)		
18-29	280	8.0 (6.7–9.4)
30-49	1097	26.5 (24.3–28.8)
50-69	1348	39.5 (37.0–41.9)
70 or more	972	26.1 (23.9–28.4)
Gender		
Female	1540	39.5 (37.1–42.0)
Male	2157	60.5 (58.0–62.9)
Ethnicty		
Non native	2061	65.3 (62.9–67.6)
Native	1251	24.1 (22.1–26.3)
Afro-Peruvian	385	10.6 (9.2–12.1)
Educational level		
None or pre- school	395	7.1 (6.1–8.3)
Primary education	1279	27.8 (25.7–29.9)
Secondary education	1152	35.1 (32.7–37.6)
Higher education	871	30.0 (27.7–32.5)
Marital status		
Single	394	11.4 (9.9–13.0)
Married	2288	62.4 (59.9–64.7)
Widowed, divorced, or separated	1015	26.2 (24.0–28.6)
Health insurance		
No	879	25.6 (23.4–27.9)
Yes	2818	74.4 (72.1–76.6)
Body Mass Index		
Underweight/Normal	931	21.0 (19.0–23.1)
Overweight	1528	42.5 (40.1–45.0)
Obesity	1238	36.5 (34.1–39.0)
Physical or psychological limitation		
No	3503	95.9 (94.9–96.8)
Yes	194	4.1 (3.2–5.1)
Depressive symptoms		
No	3331	92.2 (90.8–93.4)
Yes	366	7.8 (6.6–9.2)
Diabetes		
No	3422	90.6 (88.9–92.1)
Yes	275	9.4 (7.9–11.1)
Smoking status		
No	3255	87.9 (85.9–89.6)
Yes	442	12.1 (10.4–14.1)
Binge drinking		
No	2840	75.4 (73.1–77.6)
Yes	857	24.6 (22.4–26.9)
Wealth quintile		
Richest	488	23.7 (21.5–26.1)
Rich	542	22.0 (19.7–24.4)
Middle	658	20.1 (18.1–22.2)
Poor	845	19.0 (17.2–20.9)
Poorest	1164	15.3 (14.0–16.6)
Area of residence		
Urban	2440	84.3 (83.0–85.5)
Rural	1257	15.7 (14.5–17.0)
Natural region of residence		
Jungle	749	10.1 (9.1–11.2)
Andean	1264	19.2 (17.6–20.9)
Coast	1684	70.7 (68.7–72.6)
Altitude of residence (meters)		
0-499	1916	72.9 (70.9–74.9)
500-1499	422	6.4 (5.3–7.8)
1500-2999	567	10.0 (8.8–11.3)
3000 or more	792	10.7 (9.5–12.0)

ENDES: Encuesta Demográfica y de Salud Familiar; SD: standard deviation; CI: confidence interval.

∗ The weighting factor and sample specifications of ENDES were included.

Blood pressure measurements compatible with undiagnosed hypertension were found in 67.2% of the population. Adults in the age group of 18–29 years (94.5%) and of 70 years or older (47.5%) presented the highest and lowest prevalence of undiagnosed hypertension, respectively. Men presented a higher prevalence of undiagnosed hypertension (men: 74.7%; women: 55.7%; p <0.001). According to ethnicity, similar values of undiagnosed hypertension were found (non native: 65.9%; native: 71.1%; afro-peruvian: 66.7%). People with a higher educational level had the highest prevalence of undiagnosed hypertension (72.0%). According to marital status, single men presented the highest prevalence of undiagnosed hypertension (81.0%). The richest had the lowest prevalence of undiagnosed hypertension (62.0%). In relation to the place of residence, those in rural areas (71.7%), in the Andean region (70.4%), and those living at more than 3000 MASL (75.1%) presented the highest prevalence of undiagnosed hypertension (Table 2).

**Table 2:** Prevalence of undiagnosed hypertension among adults according to background characteristics (ENDES 2019).

Characteristic	Undiagnosed hypertension		P-value∗∗
	No (n = 1148) %∗ (95% CI)	Yes (n = 2549) %∗ (95% CI)	
Total	32.8 (30.4–35.3)	67.2 (64.7–69.6)	--
Standardized prevalence#	19.5 (17.7–21.4)	80.5 (78.6–82.3)	
Age group (years)			
18-29	5.5 (2.6–11.2)	94.5 (88.8–97.4)	<0.001
30-49	18.0 (14.5–22.1)	82.0 (77.9–85.5)	
50-69	35.2 (31.5–39.2)	64.8 (60.8–68.5)	
70 or more	52.5 (47.3–57.6)	47.5 (42.4–52.7)	
Gender			
Female	44.3 (40.3–48.4)	55.7 (51.6–59.7)	<0.001
Male	25.3 (22.4–28.3)	74.7 (71.7–77.6)	
Ethnicty			
Non native	34.1 (31.1–37.3)	65.9 (62.7–68.9)	0.156
Native	28.9 (24.8–33.5)	71.1 (66.5–75.2)	
Afro-Peruvian	33.3 (27.0–40.2)	66.7 (59.8–73.0)	
Educational level			
None or elementary school	37.5 (30.4–45.2)	62.5 (54.8–69.6)	<0.001
Primary education	41.4 (37.2–45.8)	58.6 (54.2–62.8)	
Secondary education	29.1 (25.4–33.2)	70.9 (66.8–74.6)	
Higher education	28.0 (23.7–32.8)	72.0 (67.2–76.3)	
Marital status			
Single	19.0 (14.0–25.4)	81.0 (74.6–86.0)	<0.001
Married	31.6 (28.6–34.7)	68.4 (65.3–71.4)	
Widowed, divorced, or separated	41.7 (37.0–46.6)	58.3 (53.4–63.0)	
Health insurance			
No	22.2 (18.2–26.8)	77.8 (73.2–81.8)	<0.001
Yes	36.5 (33.7–39.3)	63.5 (60.7–66.3)	
Body Mass Index			
Underweight/Normal	28.2 (23.6–33.4)	71.8 (66.6–76.4)	0.039
Overweight	31.9 (28.5–35.6)	68.1 (64.4–71.5)	
Obesity	36.5 (32.4–40.7)	63.5 (59.3–67.6)	
Physical or psychological limitation			
No	32.1 (29.7–34.5)	67.9 (65.5–70.3)	0.002
Yes	50.5 (38.5–62.4)	49.5 (37.6–61.5)	
Depressive symptoms			
No	31.4 (29.0–33.9)	68.6 (66.1–71.0)	<0.001
Yes	49.1 (40.7–57.5)	50.9 (42.5–59.3)	
Diabetes			
No	29.2 (26.9–31.7)	70.8 (68.3–73.1)	<0.001
Yes	67.1 (58.4–74.7)	32.9 (25.3–41.6)	
Smoking status			
No	34.4 (31.8–37.1)	65.6 (62.9–68.2)	0.002
Yes	21.2 (15.3–28.6)	78.8 (71.4–84.7)	
Binge drinking			
No	36.6 (33.8–39.5)	63.4 (60.5–66.2)	<0.001
Yes	21.1 (17.1–25.7)	78.9 (74.3–82.9)	
Wealth quintile			
Richest	38.0 (32.3–44.1)	62.0 (55.9–67.7)	0.019
Rich	35.9 (30.6–41.6)	64.1 (58.4–69.4)	
Middle	30.4 (25.5–35.8)	69.6 (64.2–74.5)	
Poor	29.3 (25.1–33.8)	70.7 (66.2–74.9)	
Poorest	27.8 (24.5–31.3)	72.2 (68.7–75.5)	
Area of residence			
Urban	33.6 (30.9–36.5)	66.4 (63.5–69.1)	0.019
Rural	28.3 (25.0–31.8)	71.7 (68.2–75.0)	
Natural region of residence			
Jungle	35.9 (31.6–40.5)	64.1 (59.5–68.4)	0.135
Andean	29.6 (26.3–33.2)	70.4 (66.8–73.7)	
Coast	33.2 (30.1–36.5)	66.8 (63.5–69.9)	
Altitude of residence (meters)			
0-499	33.7 (30.6–36.8)	66.3 (63.2–69.4)	0.028
500-1499	33.6 (26.7–41.4)	66.4 (58.6–73.3)	
1500-2999	34.5 (29.6–39.8)	65.5 (60.2–70.4)	
3000 or more	24.9 (20.8–29.5)	75.1 (70.5–79.2)	

Data are displayed as weighted % of the row unless otherwise indicated.

ENDES: Encuesta Demográfica y de Salud Familiar.

∗ The weighting factor and sample specifications of ENDES were included.

∗∗ Estimated P-value using the Chi-square test with Rao-Scott adjustment.

# According to the WHO population. CI: confidence interval.

In the adjusted analysis, it was found that men had a higher probability of presenting undiagnosed hypertension (aPR = 1.15; 95% CI: 1.05–1.26). Regarding the place of residence, people from the Coast had a greater probability of presenting undiagnosed hypertension (aPR = 1.17; 95% CI: 1.07–1.28). In relation to the altitude above sea level, adults who lived at 3000 MASL or more had a higher prevalence of undiagnosed hypertension (aPR = 1.20; 95% CI: 1.01–1.42) compared to those living at between 0 to 499 MASL. Furthermore, according to the wealth quintile, the poorest had a higher probability of undiagnosed hypertension compared to the richest (aPR: 1.18; 95%CI: 1.02–1.36). On the other hand, belonging to the age groups of 50–69 years (aPR = 0.79; 95% CI: 0.73–0.87) and 70 or more (aPR = 0.58; 95% CI: 0.50–0.68), represented a lower probability of presenting undiagnosed hypertension (Table 3). No association was found between sociodemographic characteristics such as ethnicity, educational level, marital status, and urban or rural area of residence and the presence of undiagnosed hypertension (Table 3). In the sensitivity analysis (using only the second blood pressure measurement to identify the presence of undiagnosed hypertension), except for the wealth quintile which was not associated with undiagnosed hypertension (p>0.05 for all quintiles), the aforementioned variables that were found to be associated with undiagnosed hypertension in the primary study analysis were also associated with this dependent variable (Table 4).

**Table 3:** Factors associated with undiagnosed hypertension among adults in Peru (ENDES 2019).

Variable	Crude		Adjusted∗	
	PR (95% CI)	P-value	aPR (95% CI)	P-value
Age group (years)				
18-29	Reference		Reference	
30-49	0.87 (0.81–0.93)	<0.001	0.94 (0.86–1.02)	0.119
50-69	0.69 (0.64–0.74)	<0.001	0.79 (0.73–0.87)	<0.001
70 or more	0.50 (0.45–0.56)	<0.001	0.58 (0.50–0.68)	<0.001
Gender				
Female	Reference		Reference	
Male	1.34 (1.23–1.46)	<0.001	1.15 (1.05–1.26)	0.002
Ethnicity				
Non native	Reference		Reference	
Native	1.08 (1.00–1.17)	0.053	1.04 (0.96–1.13)	0.356
Afro-Peruvian	1.01 (0.91–1.13)	0.820	0.95 (0.96–1.05)	0.312
Educational level				
None or elementary school	Reference		Reference	
Primary education	0.94 (0.81–1.08)	0.363	0.88 (0.76–1.02)	0.087
Secondary education	1.13 (0.99–1.29)	0.061	0.90 (0.77–1.06)	0.195
Higher education	1.15 (1.01–1.32)	0.040	0.94 (0.79–1.12)	0.480
Marital status				
Single	Reference		Reference	
Married	0.85 (0.78–0.92)	<0.001	1.00 (0.92–1.10)	0.956
Widowed, divorced or separated	0.72 (0.65–0.80)	<0.001	1.01 (0.89–1.13)	0.911
Health insurance				
No	Reference		Reference	
Yes	0.82 (0.76–0.88)	<0.001	0.92 (0.86–0.99)	0.022
Body Mass Index				
Underweight/Normal	Reference		Reference	
Overweight	0.95 (0.87–1.03)	0.218	0.91 (0.83–1.00)	0.049
Obesity	0.88 (0.81–0.97)	0.010	0.84 (0.76–0.93)	0.001
Physical or psychological limitation				
No	Reference		Reference	
Yes	0.73 (0.57–0.93)	0.012	0.89 (0.69–1.14)	0.348
Depressive symptoms				
No	Reference		Reference	
Yes	0.74 (0.63–0.88)	0.001	0.86 (0.73–1.00)	0.054
Diabetes				
No	Reference		Reference	
Yes	0.47 (0.36–0.60)	<0.001	0.55 (0.43–0.70)	<0.001
Smoking status				
No	Reference		Reference	
Yes	1.20 (1.09–1.32)	<0.001	1.02 (0.93–1.13)	0.631
Binge drinking				
No	Reference		Reference	
Yes	1.25 (1.16–1.34)	<0.001	1.03 (0.96–1.11)	0.405
Wealth quintile				
Richest	Reference		Reference	
Rich	1.03 (0.91–1.18)	0.613	1.05 (0.93–1.19)	0.399
Middle	1.12 (1.00–1.27)	0.057	1.10 (0.98–1.24)	0.113
Poor	1.14 (1.02–1.28)	0.022	1.08 (0.96–1.22)	0.207
Poorest	1.17 (1.05–1.30)	0.005	1.18 (1.02–1.36)	0.027
Area of residence				
Urban	Reference		Reference	
Rural	1.08 (1.01–1.15)	0.016	1.03 (0.95–1.12)	0.435
Natural region of residence				
Jungle	Reference		Reference	
Andean	1.10 (1.01–1.20)	0.030	1.00 (0.86–1.16)	0.955
Coast	1.04 (0.96–1.13)	0.338	1.17 (1.07–1.28)	0.001
Altitude of residence (meters)				
0-499	Reference		Reference	
500-1499	1.00 (0.89–1.13)	0.997	0.99 (0.87–1.13)	0.879
1500-2999	0.99 (0.90–1.08)	0.775	1.10 (0.95–1.27)	0.211
3000 or more	1.13 (1.05–1.22)	0.001	1.20 (1.01–1.42)	0.043

Weighting factors and sample specifications of ENDES were included for all analyses.

ENDES: Encuesta Demográfica y de Salud Familiar.

CI: confidence interval; PR: prevalence ratio; aPR: adjusted prevalence ratio.

∗ Adjusted model for all the variables that resulted in a value of p <0.2 in the crude model.

**Table 4:** Factors associated with undiagnosed hypertension in a second blood pressure measurement among adults in Peru (ENDES 2019).

Variable	Crude		Adjusted∗	
	PR (95% CI)	P-value	aPR (95% CI)	P-value
Age group (years)				
18-29	Reference		Reference	
30-49	0.87 (0.81–0.92)	<0.001	0.95 (0.87–1.03)	0.210
50-69	0.68 (0.64–0.74)	<0.001	0.80 (0.73–0.88)	<0.001
70 or more	0.50 (0.44–0.56)	<0.001	0.58 (0.50–0.68)	<0.001
Gender				
Female	Reference		Reference	
Male	1.33 (1.23–1.45)	<0.001	1.16 (1.06–1.27)	0.001
Educational level				
None or elementary school	Reference		Reference	
Primary education	0.93 (0.81–1.07)	0.289	0.88 (0.76–1.02)	0.084
Secondary education	1.10 (0.97–1.26)	0.135	0.89 (0.75–1.04)	0.141
Higher education	1.15 (1.00–1.31)	0.042	0.92 (0.77–1.10)	0.367
Marital status				
Single	Reference		Reference	
Married	0.83 (0.76–0.90)	<0.001	0.98 (0.90–1.08)	0.713
Widowed, divorced or separated	0.71 (0.64–0.80)	<0.001	1.00 (0.88–1.12)	0.954
Health insurance				
No	Reference		Reference	
Yes	0.83 (0.77–0.89)	<0.001	0.93 (0.87–0.99)	0.025
Body Mass Index				
Underweight/Normal	Reference		Reference	
Overweight	0.95 (0.87–1.04)	0.263	0.92 (0.84–1.02)	0.104
Obesity	0.88 (0.80–0.97)	0.008	0.84 (0.76–0.94)	0.002
Physical or psychological limitation				
No	Reference		Reference	
Yes	0.74 (0.57–0.95)	0.018	0.90 (0.69–1.17)	0.434
Depressive symptoms				
No	Reference		Reference	
Yes	0.78 (0.66–0.91)	0.002	0.87 (0.75–1.01)	0.064
Diabetes				
No	Reference		Reference	
Yes	0.46 (0.36–0.60)	<0.001	0.55 (0.43–0.71)	<0.001
Smoking status				
No	Reference		Reference	
Yes	1.22 (1.11–1.34)	<0.001	1.04 (0.94–1.15)	0.440
Binge drinking				
No	Reference		Reference	
Yes	1.24 (1.15–1.34)	<0.001	1.03 (0.95–1.11)	0.449
Wealth quintile				
Richest	Reference		Reference	
Rich	1.03 (0.90–1.18)	0.663	1.06 (0.94–1.21)	0.337
Middle	1.10 (0.97–1.25)	0.134	1.08 (0.95–1.23)	0.222
Poor	1.15 (1.02–1.29)	0.023	1.08 (0.95–1.23)	0.223
Poorest	1.16 (1.04–1.30)	0.008	1.15 (0.99–1.33)	0.075
Area of residence				
Urban	Reference		Reference	
Rural	1.08 (1.01–1.15)	0.022	1.01 (0.95–1.10)	0.826
Natural region of residence				
Jungle	Reference		Reference	
Andean	1.11 (1.02–1.20)	0.014	1.02 (0.88–1.18)	0.789
Coast	1.02 (0.94–1.11)	0.677	1.13 (1.04–1.24)	0.006
Altitude of residence (meters)				
0-499	Reference		Reference	
500-1499	1.01 (0.89–1.14)	0.884	1.00 (0.87–1.14)	0.965
1500-2999	1.01 (0.93–1.11)	0.757	1.08 (0.94–1.25)	0.278
3000 or more	1.17 (1.08–1.26)	<0.001	1.21 (1.02–1.42)	0.027

Weighting factors and sample specifications of ENDES were included for all analyses.

ENDES: Encuesta Demográfica y de Salud Familiar.

CI: confidence interval; PR: prevalence ratio; aPR: adjusted prevalence ratio.

∗ Adjusted model for all the variables that resulted in a value of p <0.2 in the crude model.

The adjusted analysis found that having health insurance (aPR = 0.92; 95% CI: 0.86–0.99), being obese (aPR = 0.84; 95% CI: 0.76–0.93) and having diabetes mellitus (aPR = 0.55; 95% CI: 0.43–0.70) were associated with a lower prevalence of undiagnosed hypertension (Table 3). Other characteristics such as having a physical or psychological limitation, having depressive symptoms, and consumption patterns such as smoking or binge drinking were not associated with the presence of undiagnosed hypertension (Table 3). In the sensitivity analysis, the same variables related to health status were found to be associated with undiagnosed hypertension when only the second blood pressure measurement was used to identify the presence of undiagnosed hypertension (Table 4).

**Figure 2:**
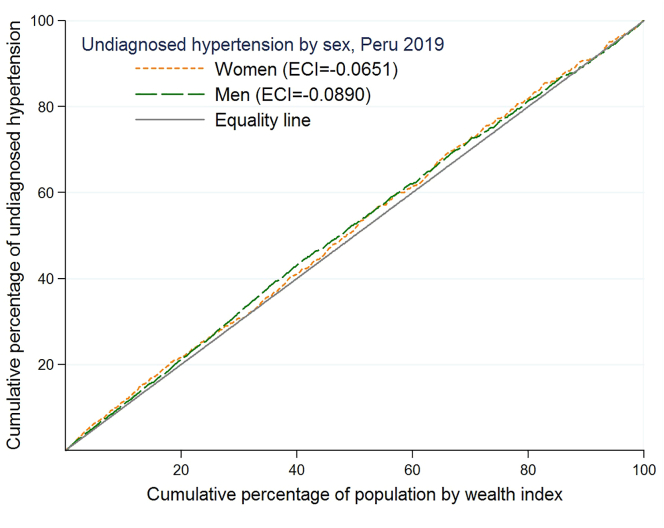
Concentration curve of undiagnosed hypertension by sex in Peru (ENDES 2019).

Figure 2 shows the CC of undiagnosed hypertension for men and women. Both curves are above the equality line, indicating a higher concentration of undiagnosed hypertension among poor men and women. The ECI for the total population was -0.0846. The negative sign of the ECI indicates inequality to the detriment of the poor concerning undiagnosed hypertension. No significant differences were reported in the ECI according to sex (p = 0.673) or according to the area of residence (p = 0.731). However, higher ECI values (greater inequality) were reported in people from the Jungle compared to people living in the Andean and the Coast regions (p = 0.004) (Table 5).

**Table 5:** Summary of results in Erreygers concentration index.

Variable	ECI	SE	P-value
General population	-0.0846	0.0269	
Gender			
Female	-0.0651	0.0460	0.673∗
Male	-0.0890	0.0330	
Area of residence			
Urban	-0.0765	0.0313	0.731∗
Rural	-0.0591	0.0397	
Natural Region			
Jungle	-0.1367	0.0504	0.004∗∗
Andean	-0.1048	0.0442	
Coast	-0.0783	0.0357	

ECI: Erreygers Concentration Index.

SE: Standard error.

∗ Z stat for differences of Erreygers Concentration Index.

∗∗ F-stat for differences of Erreygers Concentration Index.

## 4 Discussion

This study sought to determine the prevalence and factors associated with undiagnosed hypertension in the Peruvian population. It was found that more than 50% of adults with blood pressure values compatible with hypertension had no prior diagnosis. Non-diagnosis of hypertension was more frequent in men. Similarly, it was more frequent in people residing on the coast and in those who lived at more than 3000 MASL. On one hand, people over the age of 50 were less likely to have undiagnosed hypertension. Likewise, health-related characteristics such as having health insurance, being obese, and having diabetes mellitus were associated with a lower prevalence of undiagnosed hypertension. On the other hand, a higher concentration of non-diagnosis of hypertension was found in the poorest population, which would indicate the existence of inequality in the non-diagnosis of hypertension according to socioeconomic conditions.

Hypertension is also known as the “silent disease” since a proportion of the people who present this clinical condition are unaware of their status [25]. This expression is a good description of the scenario of patients with hypertension in many regions of the world and in countries such as Peru. In low-and middle-income countries, it has been reported that the prevalence of undiagnosed hypertension is between 12% and 50% [6, 8, 26, 27]. The value found in Peru is above this range, with more than half of adults with hypertension presenting undiagnosed hypertension, making this an important public health problem. Likewise, inequality in the non-diagnosis of hypertension was most concentrated in the poorest population, presenting a higher prevalence of undiagnosed hypertension compared to the wealthiest population. In the literature, it is described that the poorest population has higher blood pressure values, and given their vulnerable condition to the presence of diseases, this population requires prioritization to avoid the impact of hypertension complications on their health [28, 29]. Delayed diagnosis of hypertension leads to problems such as increased cardiovascular risk [30]. In addition, hypertension is one of the main causes of premature death in the world, with an increase from 95.9 million to 143.0 million in Disability Adjusted Life Years (DALYs) by this cause in recent years [31]. For this reason, initiatives, such as the World Hypertension Day, seek to raise awareness of the problem of hypertension in the world, to promote the incorporation of healthy lifestyles, and empower people in their health and compliance with treatment [32], given that in Peru and other regions of the world, low knowledge about hypertension is described as being related to poor control of the disease [33].

Men presented a higher prevalence of undiagnosed hypertension. In relation to sex and hypertension, in general, it is described that the proportion of men with hypertension is higher than that of women [34]. In the Peruvian population, a higher prevalence of hypertension has also been reported in men [35, 36]. The higher probability of having undiagnosed hypertension in men could be explained by their lower awareness of hypertension as described in the literature [37, 38]. In addition, it is reported that men have less adequate control of hypertension and blood pressure, which would explain the higher proportion of complications and death from cerebrovascular diseases in men, with hypertension leading among the main risk factors for death in the world. On the other hand, having health insurance was found to be associated with a lower probability of presenting undiagnosed hypertension. This result is in line with previous studies indicating that not having health insurance increases the probability of not having blood pressure checks and not having controlled blood pressure in patients with hypertension [39], as well as decreasing the probability of receiving medication to control blood pressure [40]. On the other hand, no differences were found in the proportion of people with undiagnosed hypertension according to their educational level despite the population with a higher educational level in Peru presenting higher proportions of insurance and health care compared to the poorest, including having private health insurance [41]. Hence, the provision of health insurance to the general population would be of significant value to reduce the prevalence of undiagnosed hypertension.

We reported that adults over the age of 50 or more had a lower prevalence of undiagnosed hypertension compared to younger adults (18–29 years). It was found that nine out of ten people with hypertension under the age of 30 are unaware of their problem. In general terms, an increase in age is associated with a greater probability of presenting chronic diseases such as hypertension, and a higher prevalence of hypertension has been described with increasing age in Peru [42]. Due to the demographic transition phenomenon, there has been an increase in the elderly population in various regions and countries of the world, including Peru. The clinical management of elderly patients with hypertension represents a challenge for health professionals due to conditions such as frailty, the presence of comorbidities, and the degree of functionality of the patient [43]. Late diagnosis of hypertension can lead to the presence of complications in this population, which could simultaneously have other chronic conditions that can decompensate or produce greater complications in relation to hypertension, as well as affect the effectiveness of treatment to control blood pressure [43, 44]. Although it is described that one-third of the older Peruvian adult population has hypertension, one-third of these cases do not receive treatment [45], and therefore, despite this age group having the lowest prevalence of undiagnosed hypertension, a significant proportion of this group does not receive treatment and could present complications derived from the disease. Likewise, the fact that almost all individuals under the age of 30 are unaware that they have hypertension indicates that this subgroup of the population will present a long time exposure to the disease without receiving treatment, thereby increasing the risk of presenting cardiovascular events throughout their lives [46]. Therefore, integrated strategies are required to achieve total diagnostic rates, oriented according to age groups, as well as greater coverage of disease treatment.

The place of residence was found to be associated with the prevalence of undiagnosed hypertension, considering that individuals residing in the Coastal region and at more than 3000 MASL had the highest prevalence of hypertension. In general, it has been described that the population of the Coast of Peru presents a higher prevalence of hypertension [36, 47, 48], likely because a greater proportion of the Peruvian population lives in the Coast region and due to the lifestyles of the inhabitants of this geographical region (which includes the large cities of the country). The lower diagnosis of hypertension in people who reside at more than 3000 MASL could be explained by the lack of availability of health care facilities as well as geographical barriers in the access to these facilities [49, 50]. It is postulated that altitude could play a role in the development of hypertension from exposure to chronic hypoxia presented in inhabitants of high altitude regions [51]. Considering all of the above, programs that seek to promote early diagnosis and treatment of patients with hypertension are needed in the populations with these characteristics of residence in order to identify and reduce the number of untreated patients with hypertension.

Health conditions such as being obese and having diabetes mellitus were found to be related to a lower probability of presenting undiagnosed hypertension. The biological mechanisms related to a higher probability of obese patients developing hypertension are currently under study, including the influence of visceral fat and excessive weight gain [52, 53, 54]. Likewise, it has been reported that patients with hypertension have a greater probability of presenting comorbidities compared to those without this disease [55, 56], and also patients with hypertension and comorbidities are more likely to present adequate control of hypertension [57]. This is in line with and could explain our findings in that adults presenting comorbidities are more likely to receive medical care, either due to complications derived from hypertension or from these comorbidities, thereby increasing the probability of blood pressure being diagnosed and controlled in these patients. Furthermore, the association between the presence of diabetes mellitus and hypertension has been widely described, including studies that report this finding in the Peruvian population [35]. Considering the positive trends in the population with overweight/obesity and diabetes in Peru and worldwide [58], an increase in cases with hypertension and these comorbidities is expected, and therefore, efforts to identify the presence of hypertension in patients with these clinical conditions should be prioritized to reduce hypertension-related complications.

Among the limitations of this study, the cross-sectional study design limits the possibility of studying causal relationships among the factors studied and the presence of undiagnosed hypertension. Additionally, a secondary analysis of the database was performed, and the data analyzed may not be accurate due to recall bias, inadequate understanding of some questions, or inaccurate measurements in the survey. In addition, the ENDES does not record data on diseases, risk factors, or treatments that could modify blood pressure. Likewise, given the differences between the first and second blood pressure measurements in the ENDES, it would be important to have a third measurement. On the other hand, if the American College of Cardiology/American Heart Association 2017 guidelines are applied, it is expected that the prevalence of hypertension and undiagnosed hypertension will increase significantly [59]. However, the authors consider that the use of a nationally representative database allows the study of a highly prevalent problem such as hypertension, as well as identification of the factors associated with the non-diagnosis of this chronic condition, and consider these findings useful from the Peruvian and global perspective, to continue with the study of the non-diagnosis of hypertension and its related factors.

In conclusion, it was found that hypertension remains undiagnosed in at least one out of every two Peruvian adults. Sociodemographic and health-related factors were found to be associated with a greater or lesser probability of having undiagnosed hypertension. Additionally, there is inequality in the non-diagnosis of hypertension, with non-diagnosis being concentrated in the poorest population. Thus, in a significant proportion of Peruvian adults, hypertension is not diagnosed and therefore not treated, and this may affect their current and future health status. Based on the results of our study, it is possible to identify some population subgroups such as men as well as residents of the Coast and inhabitants of communities at greater than 3000 MASL in which interventions for screening and treatment of hypertension should be prioritized. Likewise, it is necessary to study the inequalities in the non-diagnosis of hypertension, focused on the poorest populations and those most vulnerable to the complications of this disease.

## Declarations

### Author contribution statement

Delia Vanessa Guerrero-Díaz, Wency Cecilia Montoya-Rivera, Carlos Rojas-Roque, Guido Bendezu-Quispe: Analyzed and interpreted the data; Wrote the paper.

Manuel Alberto Chacón Díaz: Conceived and designed the experiments; Performed the experiments.

Akram Hernández-Vásquez: Conceived and designed the experiments; Performed the experiments; Analyzed and interpreted the data; Wrote the paper.

### Funding statement

This research did not receive any specific grant from funding agencies in the public, commercial, or not-for-profit sectors.

### Data availability statement

Data associated with this study has been deposited at http://iinei.inei.gob.pe/microdatos/

### Declaration of interests statement

The authors declare no conflict of interest.

### Additional information

This study will serve as a partial requirement for two of the authors to obtain their medical degree (Delia Vanessa Guerrero-Díaz and Wency Cecilia Montoya-Rivera).
